# Mental Hospital Matters

**Published:** 1911-11-04

**Authors:** G. Stevens Pope

**Affiliations:** late medical superintendent of the Somerset and Bath Asylum, Wells


### MENTAL HOSPITAL MATTERS.

Dr. G. Stevens Pope, late medical superintendent of the Somerset and Bath Asylum, Wells, writes : I have drawn the attention of the staff to your paper. I should be sorry, however, to see it made the medium of ventilating discontent or complaints, as there is other machinery in existence. I would also deprecate the wild statements made before the Select Committee of the Commons recently sitting on the "Asylum Officers' Superannuation and Employment" Bill by certain malcontents who injure our cause. As far as this Asylum is concerned, my Committee is a generous, enlightened one, and we have no grievances.

[In congratulating Dr. Pope upon having so excellent a Committee, we beg to assure him that, as our article last week indicates, the fullest editorial discretion, with judicial care, will be exercised.—Ed. The Hospital.]

